# miR-17-92 cluster in osteoarthritis: Regulatory roles and clinical utility

**DOI:** 10.3389/fgene.2022.982008

**Published:** 2022-11-29

**Authors:** Xuefeng Pan, Xiao Cen, Xiner Xiong, Zhihe Zhao, Xinqi Huang

**Affiliations:** ^1^ State Key Laboratory of Oral Diseases and National Clinical Research Center for Oral Diseases, West China Hospital of Stomatology, Sichuan University, Chengdu, China; ^2^ Department of Orthodontics, West China Hospital of Stomatology, Sichuan University, Chengdu, China; ^3^ Department of Temporomandibular Joint, West China Hospital of Stomatology, Sichuan University, Chengdu, China; ^4^ Hospital of Stomatology, Zunyi Medical University, Zunyi, China

**Keywords:** osteoarthritis, miRNA, chondrocyte apoptosis, extracellular matrix degradation, bone remodeling, synovitis

## Abstract

Osteoarthritis (OA) is the most prevalent articular disease, especially in aged population. Caused by multi-factors (e.g., trauma, inflammation, and overloading), OA leads to pain and disability in affected joints, which decreases patients’ quality of life and increases social burden. In pathophysiology, OA is mainly characterized by cartilage hypertrophy or defect, subchondral bone sclerosis, and synovitis. The homeostasis of cell–cell communication is disturbed as well in such pro-inflammatory microenvironment, which provides clues for the diagnosis and treatment of OA. MicoRNAs (miRNAs) are endogenous non-coding RNAs that regulate various processes *via* post-transcriptional mechanisms. The miR-17-92 cluster is an miRNA polycistron encoded by the host gene called MIR17HG. Mature miRNAs generated from MIR17HG participate in biological activities such as oncogenesis, neurogenesis, and modulation of the immune system. Accumulating evidence also indicates that the expression level of miRNAs in the miR-17-92 cluster is tightly related to the pathological processes of OA, such as chondrocyte apoptosis, extracellular matrix degradation, bone remodeling, and synovitis. In this review, we aim to summarize the roles of the miR-17-92 cluster in the underlying molecular mechanism during the development and progression of OA and shed light on the new avenue of the diagnosis and treatment of OA.

## Introduction

Osteoarthritis (OA) is the most prevalent joint disease, which is becoming an increasing social-economic problem worldwide (Bortoluzzi et al., 2018; Peat and Thomas, 2021). It has been estimated that over 3% people are troubled with symptomatic OA in the world (Katz et al., 2021). Clinically, to relieve pain and joint disability, two leading symptoms caused by inflammatory microenvironment and articular tissue destruction, a comprehensive plan for OA management is required (Jang et al., 2021; Sharma, 2021). Patient education and lifestyle change should be applied first if necessary (Hunter and Bierma-Zeinstra, 2019). Pharmaceutical therapy (e.g., nonsteroidal anti-inflammatory drugs (NSAIDs), hyaluronic acid, protease inhibitors, and bisphosphonates), *via* oral medication or intra-articular injection, could alleviate the pain and promote tissue repair to some extent (Conaghan et al., 2019; Jones et al., 2019; Latourte et al., 2020). If the lesions advance progressively and the aforementioned conservative methods do not work, total joint replacement might be the only choice currently (Ferguson et al., 2018; Price et al., 2018).

MicroRNAs (miRNAs) are a class of endogenous non-coding RNAs with about 22 nucleotides in length, which regulate the expression of genes *via* the post-transcriptional mechanism (Bartel, 2004). Numerous functions have been ascribed to miRNAs in various physiological and pathological activities during these decades (Liu et al., 2014; Galagali and Kim, 2020). For example, miR-125, mammalian ortholog of the first reported animal miRNA lin-4, was found to regulate cancers and cardiovascular and cerebrovascular diseases (Peng et al., 2021; Wang et al., 2021). The well-known let-7 was originally found in *Caenorhabditis elegans* by Reinhart et al. (2000), following which researchers identified that this miRNA is highly evolutionarily conserved across species and functions in tumor suppressing, organism development, and stem cell differentiation (Thornton and Gregory, 2012; Ventayol et al., 2014; Lee et al., 2016). The biogenesis of miRNA is a complicated and precisely regulated process (Lin and Gregory, 2015; Lee et al., 2016; Ali Syeda et al., 2020). In brief, primary miRNA (pri-miRNA) is first synthesized in the nucleus with a hairpin stem–loop structure. The Drosha/DGCR8 complex cleaves the pri-miRNA into a 60–70-nucleotide pre-miRNA, and exportin 5 subsequently translocates the pre-miRNA into cytoplasm. In cytoplasm, pre-miRNA is processed into a double-stranded RNA (dsRNA) with ∼22 base pairs in length by Dicer. One strand of the dsRNA is loaded on the Argonaute (AGO) to form an RNA-induced silencing complex (RISC), which has the potential to regulate various biological processes by binding to the 3′ untranslated region (3′UTR) of target genes. miRNAs regulate gene expression *via* two mechanisms: suppressing protein translation by inhibiting its initiation or elongation and promoting the cleavage or degradation of mRNAs (Saliminejad et al., 2019).

miR-17-92 is a classical polycistronic cluster, and its roles in tumor and normal development were extensively studied (Chen et al., 2013; Mogilyansky and Rigoutsos, 2013; Murphy et al., 2013). A recent study also reported the relationship between miR-17-92 and OA. For example, Kong et al. (2017) collected the plasma samples of OA patients and health control for miRNA detection and analysis. miR-19b-3p and miR-92a-3p, constituents of the miR-17-92 cluster, were among the eight top differentially expressed miRNAs. In the present review, we aim to summarize the current knowledge about the underlying molecular mechanism of the miR-17–92 cluster during the development and progression of OA and shed light on the new avenue of the diagnosis and treatment of OA.

### The pathology of OA

Normally, articular cartilage coordinates with subchondral bone and synovial membrane to keep the joint in homeostasis (Zhen and Cao, 2014). The cartilage buffers external stress, while the subchondral bone transmits and absorbs the mechanical loading, and synovial fluid lubricates the joint and provides the avascular cartilage with nutrition and biological cues (Madry, 2010; Lories and Luyten, 2011). Considering their interplay under the physiological condition, it is natural to hold the belief that the progression of OA is not just questions about the cartilage. During the onset and progression of OA, loss of cartilage causes excessive loading on subchondral bone, which subsequently leads to bone lesions such as microfracture, necrosis, and fibrosis (Zhen and Cao, 2014). With the constant production and secretion of pro-inflammatory cytokines, nearby synovium, ligament, and even periarticular muscles will be affected (Katz et al., 2021).

**FIGURE 1:**
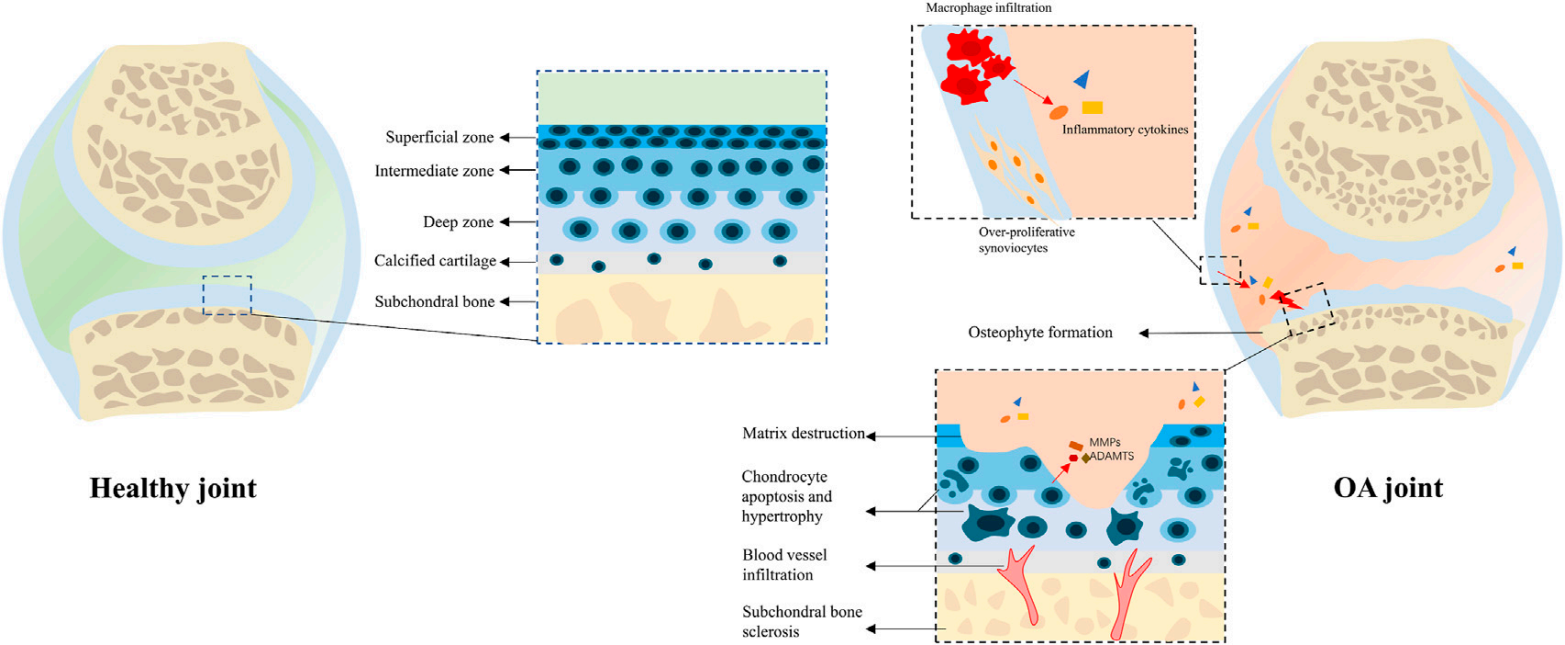
Physiological and pathological structure of the joint. In a healthy joint, the cartilage coordinates with subchondral bone, synovial membrane, and other peri-articular tissues to keep the joint in homeostasis. In an OA joint, chondrocyte hypertrophy and apoptosis occur with cartilage matrix degradation; subchondral bone changes including sclerosis, osteophyte formation, and blood vessel infiltration can also be observed; synoviocytes are over-proliferative, and plenty of macrophages infiltrate into the synovial membrane to secret pro-inflammatory cytokines, which deteriorate the microenvironment.

Mechanistically, the apoptosis and hypertrophy of chondrocyte occur during OA progression, and the normal structure of the matrix is destructed by matrix metalloproteinases (MMPs), a disintegrin and metalloproteinase with thrombospondin motifs (ADAMTS); blood vessels from bone marrow invade through the osteochondral junction into the calcified cartilage zone, leading to endochondral osteogenesis (Karsdal et al., 2014; Goldring and Goldring, 2016). In subchondral bone, bone turnover is enhanced during the early OA stage, which causes the subchondral bone becoming thinner (Hu et al., 2021). During the late stage, growth factors like transforming growth factor beta (TGF-β) and bone morphogenetic proteins (BMPs), which promote matrix production and protect joints from damage under physiological conditions, would stimulate osteophyte production and subchondral bone sclerosis under such abnormal environment (Hu et al., 2021). In synovium, chemokines are secreted to promote monocyte/macrophage infiltration, which stimulates excessive proliferation of synovial fibroblasts and production of inflammatory cytokines (Kapoor et al., 2011; Sanchez-Lopez et al., 2022). The aforementioned pathological processes of OA have been sketched in Figure 1.

## The miR-17-92 cluster and its biogenesis

**FIGURE 2:**
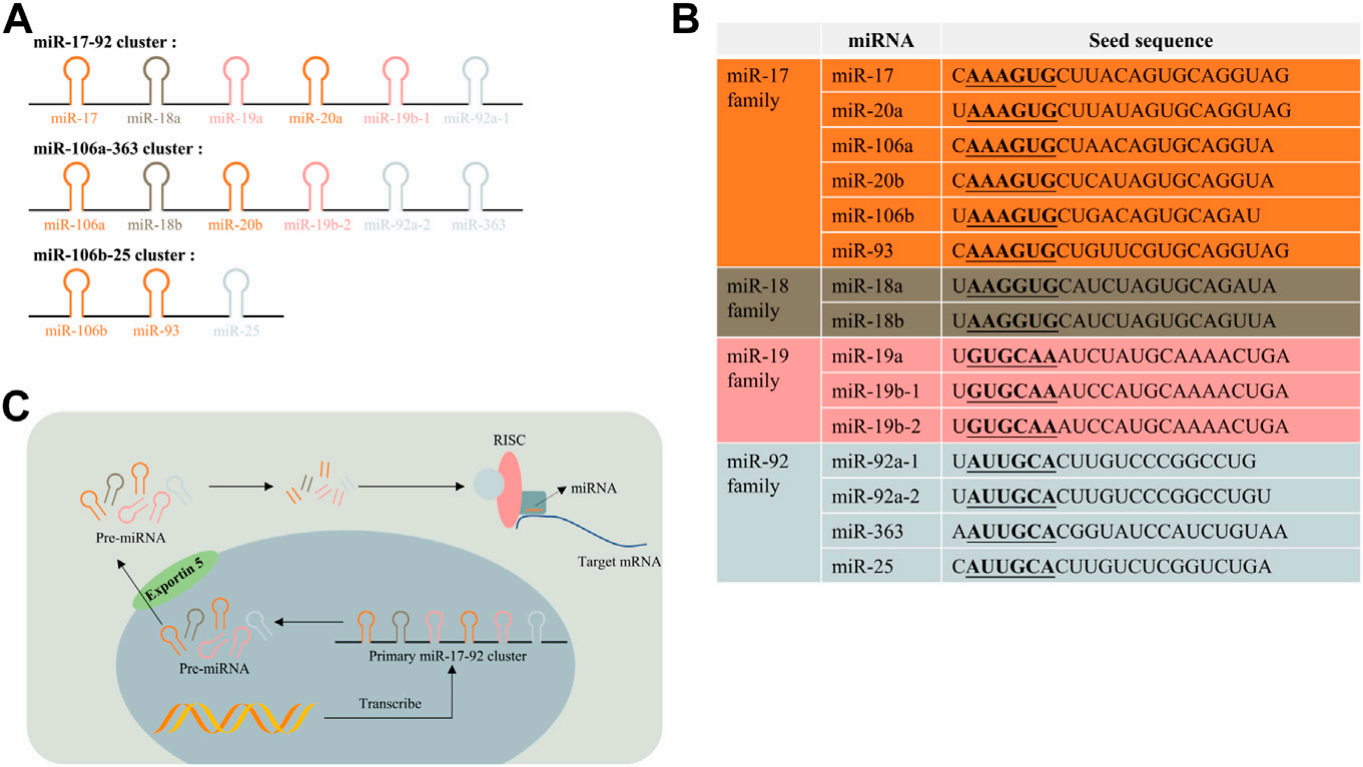
miR-17-92 cluster and their paralogs. **(A)** miR-17-92 cluster, miR-106a-363 cluster, and miR-106b-25 cluster are three paralogs in the human genome; **(B)** they can be classified into four subfamilies based on their seed sequence; **(C)** Biogenesis of the miR-17-92 cluster. RISC, RNA-induced silencing complex.

The miR-17-92 cluster (also known as oncomir-1) is an important miRNA polycistron located in the non-protein-coding region of chromosome 13 open reading frame 25 (C13orf25), which is also referred to as the miR-17-92 host gene (MIR17HG) (Mogilyansky and Rigoutsos, 2013). There are six mature miRNAs generated from the miR-17-92 cluster transcript: miR-17, miR-18a, miR-19a, miR-20a, miR-19b-1, and miR-92a-1. These miRNAs were reported to regulate biological processes including oncogenesis, neurogenesis, and immune system modulation (Mendell, 2008; Osada and Takahashi, 2011; Baumjohann, 2018). The miR-106a-363 cluster (located on chromosome X) and miR-106b-25 cluster (located on chromosome 7) are two paralogs of the miR-17-92 cluster in the human genome (Petrocca et al., 2008; Gruszka and Zakrzewska, 2018). The former cluster contains six miRNAs: miR-106a, miR-18b, miR-20b, miR-19b-2, miR-92a-2, and miR-363. The latter one contains three miRNAs: miR-106b, miR-93, and miR-25. According to their seed sequence, miRNAs in these three clusters are categorized into four groups: the miR-17 family, the miR-18, the miR-19, and the miR-92 family (see Figures 2A,B).

The biogenesis of the miR-17-92 cluster is the same as processes of other miRNAs, except there contains multiple stem–loop structures to form six miRNAs in a single transcript (Olive et al., 2010) (see Figure 2C). The production of these miRNAs could be regulated by a class of transcriptional factors. Members of the miR-17-92 and miR-106a-363 cluster were consistently observed to be upregulated when the transcription factor c-Myc was elevated in multiple cell types, and subsequent experiments validated that c-Myc could directly bind to miR-17-92 genomic locus-MIR17HG (O’Donnell et al., 2005). The amplification of another oncogenic gene n-Myc was discovered to upregulate miR-17-92 *via* transcription regulation (Schulte et al., 2008). Other transcription factors from the E2F transcription factor family (e.g., E2F1, 2, and 3) and ETS transcription factor family (e.g., Spi-1 and Fli-1) were reported to participate in the regulation of miR-17-92 biogenesis as well (Woods et al., 2007; Kayali et al., 2012). In addition, post-transcriptional factor like the tertiary structure of transcripts could also influence the biogenesis of miRNAs (Chaulk et al., 2011; Chakraborty et al., 2012). However, mechanisms of how transcription factor network and post-transcriptional factors control the biogenesis of the miR-17-92 cluster remains elucidated even with the knowledge mentioned previously.

## miR-17-92 in cartilage degradation

**FIGURE 3:**
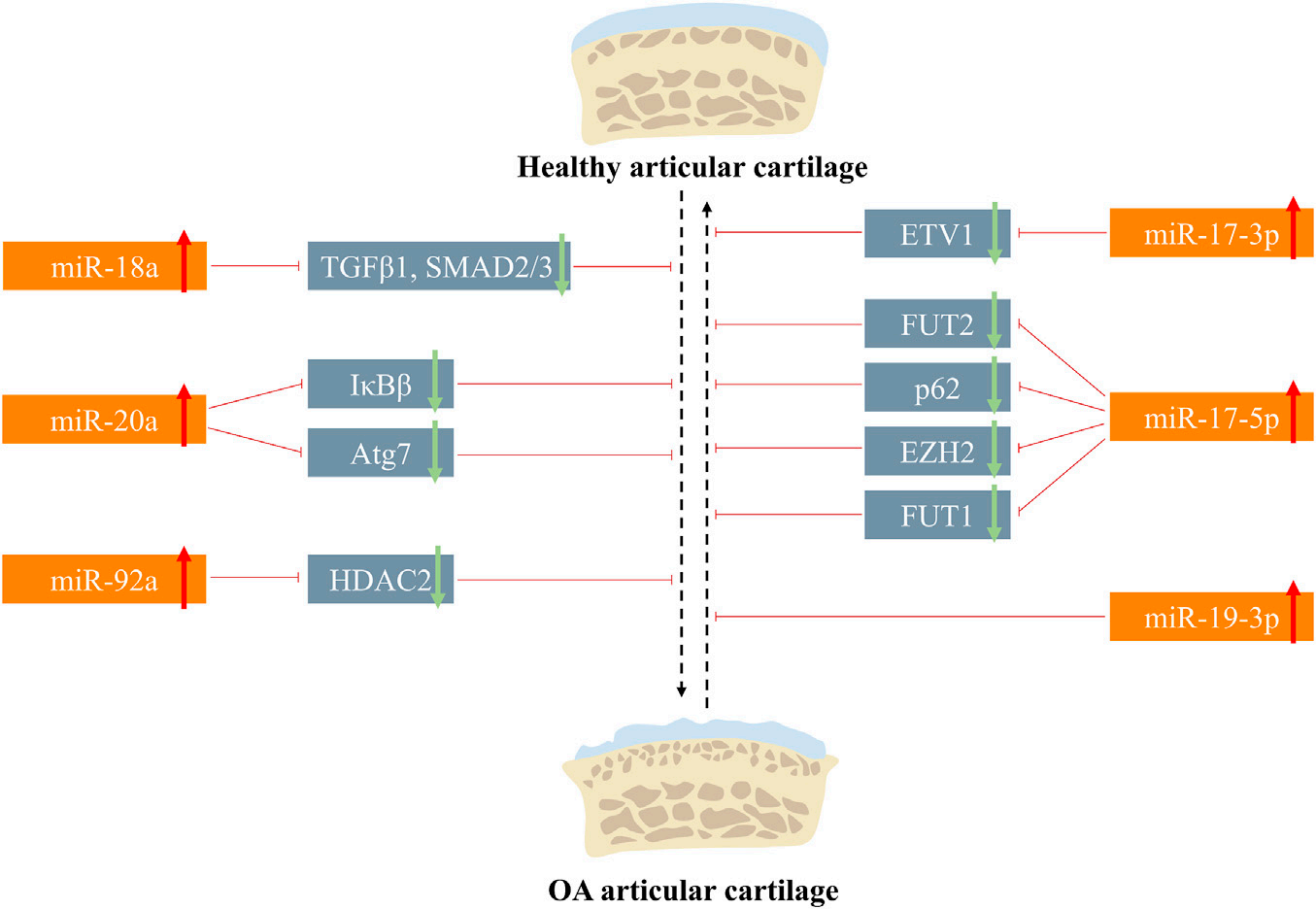
Roles of the miR-17-92 cluster in the articular cartilage metabolism.

Articular cartilage is composed of chondrocytes along with their surrounding matrix, which features the joints with the load-bearing characteristic (Guilak et al., 2018). However, the cartilage has weak ability to self-repair in response to chronic or acute pathological stimuli due to its shortage of blood supply (Makris et al., 2015; Robinson et al., 2016). Hence, proper interventions are necessary for cartilage regeneration when it is destructed. Exploring mechanisms of cartilage repair is a vital path for OA treatment (Goldring and Marcu, 2012). Interleukin-1beta (IL-1β) and lipopolysaccharide (LPS) are the two most common factors used for *in vitro* OA phenotype induction (Wang et al., 2019; Zhang H et al., 2021), and members of the miR-17-92 cluster were found to attenuate or promote OA progression in such an *in vitro* OA model. For example, miR-17a-5p, miR-19b-3p, and miR-92a-3p were reported to prevent OA progression in IL-1β-induced chondrocyte apoptosis and extracellular matrix (ECM) degradation (Mao et al., 2017a; Duan et al., 2019; Li Y et al., 2020). Under LPS induction, miR-20a inhibitor attenuated pro-inflammatory response of chondrocytes (Zhao and Gong, 2019). Based on current research, multiple signaling pathways (e.g., mitogen-activated protein kinase (MAPK) pathway, TGF-β pathway, Wnt/β-catenin, and nuclear factor kappa-B (NF-κB)) have been found to participate in the chondrocyte and ECM metabolism (van der Kraan, 2017; Choi et al., 2019; Shang et al., 2021; Li et al., 2022) (Table 1) (See Figure 3).

**TABLE 1:** Expression level of the miR-17-92 cluster and its function during OA progression.

miRNA	Expression level	Target gene	Expression level of the target gene	Function of miRNA	Upstream lncRNA	Reference
miR-18a	Downregulated in hypoxia-exosomes of BMSCs	-	-	Its knockdown relieves OA	-	Zhang Y et al. (2022)
miR-17-3p	Downregulated in LPS-induced chondrocytes	ETV1	Up	Its knockdown promotes LPS-induced C28/I2 cell injury	lnc HOTAIR	Chen et al. (2017)
miR-18a	Upregulated in the rat knee OA model	TGFβ1, SMAD2, and SMAD3	Down	Its overexpression accelerates OA progression by promoting chondrocyte hypertrophy	-	Lian et al. (2020)
miR-17-5p	Downregulated in OA cartilage	FUT2	Up	Its overexpression alleviates knee OA progression *in vivo* and suppresses IL-1β-induced ECM degradation and chondrocyte apoptosis *in vitro*	lnc HOTAIR	Hu et al. (2018)
miR-20a	Upregulated in plasma of OA patients	IκBβ	Down	Its knockdown alleviates LPS-induced chondrocyte apoptosis *in vitro*	-	Zhao and Gong (2019)
miR-17-5p	Downregulated in the mouse knee OA model	p62	Up	Its overexpression promotes autophagy in SW1353 cells	-	Li et al. (2018)
miR-20a	Downregulated during chondrogenic differentiation	Atg7	Up	Its overexpression suppresses chondrogenic differentiation of ATDC5 cells *via* inhibiting autophagy	-	Xu et al. (2019)
miR-92a-3p	Upregulated during hMSC chondrogenic differentiation and downregulated in OA cartilage	HDAC2	Down	Its overexpression increased histone acetylation of cartilage-specific genes including Col2A1 and ACAN	-	Mao et al. (2017a)
miR-17-5p	Downregulated in OA cartilage	EZH2	Up	Its overexpression inhibits IL-1β-induced ECM degradation and chondrocyte apoptosis *in vitro*	-	Li Y et al. (2020)
miR-17-5p	Downregulated in OA cartilage	FUT1	Up	Its overexpression attenuates IL-1β-induced ECM degradation and chondrocyte apoptosis *in vitro*	lnc TUG1	Li Z et al. (2020)
miR-19a-3p	Downregulated in OA cartilage	-	-	Its knockdown promotes LPS-induced chondrocyte inflammatory injury	lnc DANCR	Li et al. (2021)
miR-17-3p	Downregulated during osteogenesis	SOX6	Up	Its overexpression inhibits osteoblast differentiation	-	Chen et al. (2020)
miR-92a	Downregulated in TGF-β1-treated OA synovial fibroblasts	FOXO3	Up	Its overexpression promotes inflammatory mediator expression in OA fibroblasts	-	Kuo et al. (2019)
miR-92a-3p	Downregulated in TNF-α-treated synoviocytes	KLHL29	Up	Its knockdown promotes chemokine and inflammatory factors in synoviocytes co-cultured with micro-fragmented adipose tissue	-	Shi et al. (2022)
miR-18a-3p	-	-	-	Its overexpression inhibits synovial membrane cell proliferation and reduces inflammatory cells	-	Feng et al. (2022)
miR-18a	Downregulated in OA knee anterior cruciate ligaments	-	-	-	-	Li et al. (2017)

BMSCs, bone marrow stem cells; OA, osteoarthritis; LPS, lipopolysaccharide; ECM, extracellular matrix.

### MAPK pathway

MAPK is a class of serine/threonine-protein kinases, including three main types of subfamily members: the extracellular-signal-regulated kinases (ERKs), the c-jun N-terminal kinase (JNKs), and the p38 kinases (Lee et al., 2020; Sinkala et al., 2021). They can transfer the extracellular signals into the nucleus *via* the MAP3K–MAP2K–MAPK cascade to participate in cellular activities like proliferation, differentiation, and apoptosis (Chen et al., 2018; Ostojic et al., 2021). It was reported that bone marrow stem cell-derived extracellular vesicles (BMSC-EVs) could alleviate the IL-1β-induced chondrocyte inflammatory phenotype (Zhang B et al., 2022), and such effect was further improved with the treatment of BMSC-EV generated in a hypoxic environment. RNA sequencing showed that 12 miRNAs were aberrantly expressed in hypoxia BMSC-EV, in which miR-18a was downregulated, indicating its involvement in exosome-treated OA progress, and Kyoto Encyclopedia of Genes and Genomes (KEGG) analysis showed that such progress may be mediated by the MAPK pathway (Zhang Y et al., 2022). Chen et al. (2017) found that lipopolysaccharide (LPS) promoted cellular injury and cytokine secretion of human articular chondrocytes, which was influenced by the lncHOTAIR-miR-17-3p-ETV1 network. Knockdown of HOTAIR and ETV1 or overexpression of miR-17-3p could reverse LPS-mediated apoptosis, and subsequent results verified that the ceRNA regulatory triad regulated such process through activating MAPK signaling (Chen et al., 2017).

### TGF-β pathway

The TGF-β superfamily consists of factors including TGF-β, BMPs, and growth and differentiation factors (GDFs), which regulate gene expression in an SAMD-dependent or SMAD-independent way (Thielen et al., 2019; Cherifi et al., 2021). Accumulating evidence has shown that the TGF-β pathway is tightly related to the osteoarthritis progression and cartilage regeneration (MacFarlane et al., 2017; Wang C et al., 2020; Wang G et al., 2020). Lian et al. (2020) reported that miR-18a was significantly upregulated in IL-β-induced rat knee OA models; in *in vitro* experiments, hypertrophic genes like COL10A1 and MMP13 increased in the miR-18a overexpression group after 14 days of chondrogenic differentiation and 14 days of hypertrophic induction. As cartilage hypertrophy is a crucial step during OA progression, miR-18a has the potential to regulate OA progress negatively. Gene Ontology (GO) analysis indicated the underlying relationship between miR-18a and TGF-β; bioinformatics found that there existed binding sites of miR-18a in the 3′UTR of TGF-β1, SMAD2, and SMAD3, which was validated by RIP assay and luciferase reporter assay (Lian et al., 2020).

### Wnt/β-catenin pathway

The nomination of Wnt comes from the integrase-1 gene in mice and the wingless gene in *Drosophila*. Wnt gene regulates biological activities *via* two distinctive ways (namely, canonical pathway and noncanonical pathway). The noncanonical pathway includes the Wnt-Ca^2+^ pathway and Wnt-atypical protein kinase C pathway. The canonical pathway is dependent on β-catenin, so it is also called as the Wnt/β-catenin pathway. With regard to the Wnt/β-catenin pathway, in the absence of the extracellular Wnt signal, a compound that is comprised of axis inhibition protein (AXIN), adenomatous polyposis *coli* (APC), glycogen synthase kinase-3 (GSK-3), and casein kinase 1 (CK1) constantly degraded cytoplasmic β-catenin *via* the ubiquitination mechanism; when the ligand Wnt binds to the transmembrane receptor, cytoplasmic β-catenin will be released from the destruction complex and regulates target genes *via* translocating into the nucleus (Zhou et al., 2017; Shang et al., 2021). Except for its roles in embryonic development and cancers, the Wnt/β-catenin pathway is also tightly related to bone remodeling and cartilage homeostasis (Lietman et al., 2018; Sun J et al., 2020). Hu et al. (2018) discovered the HOTAIR–miR-17-5p–FUT2 cascade in regulating matrix degradation and cell apoptosis in IL-1β-induced experimental OA models. Due to the correlation between FUT family and Wnt/β-catenin signaling, they made the hypothesis that FUT2 participated in the Wnt/β-catenin pathway. Results showed that FUT2 upregulation significantly increased phosphorylated GSK-3β (member of the β-catenin destruction complex) expression and nuclear β-catenin accumulation, which are the signs of Wnt/β-catenin activation.

### NF-κB pathway

RelA/p65, RelB, c-Rel, NF-κB1/p105, and NF-κB2/p100 are the five constituents of the NF-κB transcription factor family (Lepetsos et al., 2019). In the resting state, NF-κB dimers are inhibited *via* binding with NF-κB inhibitors (IκBs). When the NF-κB pathway is activated, IκBs are phosphorylated and degraded by IκB kinases (IKKs) to relieve NF-κB (Taniguchi and Karin, 2018). Finally, NF-κB is translocated into the nucleus to regulate downstream gene expression. The activation of NF-κB signaling provokes cartilage matrix degradation and chondrocyte hypertrophy *via* producing catabolic mediator (e.g., MMPs and aggrecanases) and inflammatory cytokines (e.g., IL-1β and tumor necrosis factor-alpha (TNF-α)) (Olivotto et al., 2015). Chen et al. (2017) reported that miR-17-3p mediated OA phenotype regulation *via* NF-κB signaling. From another research by Zhao and Gong (2019), it was found that IκBβ, a constituent of the IκB family, could be directly targeted by miR-20a and the inhibition of miR-20a could upregulate IκBβ, thus protecting chondrocytes *via* NF-κB signaling suppression.

### Autophagy

Autophagy is a bio-protective mechanism in response to endogenous or exogenous stress (Xia et al., 2021). Intracellular components (e.g., pathogens, damaged organelles, and misfolded proteins) engulfed by autophagosomes are delivered to the lysosomal compartment for degradation and recycling (Dikic and Elazar, 2018). Three kinds of autophagy have been described currently (Mizushima and Levine, 2020; Kitada and Koya, 2021). Macroautophagy is the best understood type, where intracellular contents are first engulfed to form autophagosomes, which are fused with enzyme-enriched lysosomes to degrade contents in a selective or nonselective manner; microautophagy refers to the process in which cytoplasmic components are directly sequestered into lysosomes and chaperone-mediated autophagy selectively degrades proteins with the assistance of chaperone complexes (Mizushima and Levine, 2020; Kitada and Koya, 2021). The majority of human diseases like cancers, neurodegenerative disorders, and metabolism diseases are influenced by autophagy according to the research studies till date (Levy et al., 2017; Scrivo et al., 2018; Klionsky et al., 2021). Meanwhile, it was observed that autophagy was constantly activated under physiological articular cartilage while disturbed in aged and OA joints (Duarte, 2015; Li et al., 2016), hinting that they are tightly correlated.

Selective autophagy receptors mediate the selective degradation of aggregated proteins, dysfunctional mitochondria, and pathogens during macroautophagy. The deficiency of p62 (also referred to as sequestosome-1), a prototype autophagy receptor, leads to ubiquitinated protein accumulation and mitochondrial defects *via* impairing the selectivity of autophagy (Aparicio et al., 2020). Decreased miR-17-5p expression and suppressed autophagy were concurrently detected in the experimental mouse OA model (Li et al., 2018). The 3′-UTR of p62 was found to contain a conserved binding site with miR-17-5p, which was validated by luciferase reporter assay. However, deeper investigations on the underlying mechanisms of the miR-17-92/p62 axis and its effect on OA relief remain to be elucidated.

It was found that the miR-17-92 cluster was all decreased during ATDC5 cell chondrogenic differentiation (Xu et al., 2019). Among the six members, miR-20a showed the most significant inhibition trend on chondrogenesis. The mRFP-GFP-LC3 adenoviral vector was transfected into ATDC5 cells to trace the dynamic autophagic activities. At different induction time points (i.e., 24, 36, 48, and 72 h), increased red and yellow puncta in the chondrogenic group indicated the active state of autophagy, which can be blocked by miR-20a (Xu et al., 2019). Dozens of autophagy-related proteins were discovered in controlling the dynamics of membranes during autophagy (Xie et al., 2015). For instance, Atg5 along with Atg12/Atg16 is essential in autophagosome formation (Romanov et al., 2012; Sheng et al., 2018); Atg7 dysregulation significantly influenced the COL Ⅱ synthesis and secretion (Cinque et al., 2015). Xu et al. (2019) reported that disruption of Atg7 could reverse the promotion effect of miR-20a inhibitor on ATDC5 chondrogenic differentiation and autophagic vacuole production.

### Histone modification

Histones are alkaline proteins that envelope DNA. Histone modification (e.g., acetylation, methylation, and phosphorylation) alters the conformation of chromatin and influences transcription of genes (Stoll et al., 2018; Zhang Y et al., 2021). Histone deacetylases (HDACs) were reported to regulate chondrogenesis and cartilage degradation *via* deacetylating nucleosomal histone (Carpio and Westendorf, 2016; Meng et al., 2018). miR-92a-3p and HDAC2 were aberrantly expressed during MSC chondrogenesis and OA progression, and the luciferase reporter assay confirmed their interaction (Mao et al., 2017b). Moreover, the acetylation of histone H3 on Col Ⅱ and aggrecan promoter was enhanced by miR-92a-5p, contributing to the process of cartilage protection. Enhancer of zeste homolog 2 (EZH2), a histone methyltransferase, was upregulated in OA cartilage (Li Z et al., 2020), hinting its vital roles in OA pathogenesis. However, such effect can be attenuated by upstream miR-17-5p.

### Others

Long non-coding RNAs (lncRNAs) are a class of non-coding endogenous RNAs with over 200 nucleotides in length (Herman et al., 2022). Cytoplastic lncRNAs usually function as a miRNA sponge to regulate downstream target genes *via* classical ceRNA mechanisms (Thomson and Dinger, 2016). The roles of lncRNAs during OA-related ECM degradation and chondrocyte apoptosis were identified as well (Ghafouri-Fard et al., 2021). Hu et al. (2018) transfected chondrocytes with the HOTAIR pcDNA3.1 vector and showed that the overexpression of HOTAIR significantly promoted chondrocyte apoptosis and synthesis of cartilage-degrading enzymes (i.e., MMP13 and ADAMTS-5), while ECM-formation genes like COLⅡ and aggrecan were inhibited in the IL-1β-induced inflammatory environment. The addition of its target miR-17-5p could reverse such a pro-osteoarthritis trend *via* the FUT2-Wnt/β-catenin pathway. Another research discovered that the HOTAIR-miR-17-3p axis had the potential to influence LPS-induced OA (Chen et al., 2017). TUG1, an lncRNA related to cell apoptosis, was reported to target miR-17-5p (Li2 et al., 2020). Differentiation antagonizing non-protein coding RNA (DANCR) is an oncogenic lncRNA. Except for its vital roles in cancer progression, DANCR was reported to participate in stem cell differentiation. Li et al. (2021) found that si-DANCR could protect human chondrocytes from LPS-induced apoptosis, and miR-19a-3p was a vital mediator.

## miR-17-92 in subchondral bone remodeling

Subchondral bone refers to the cortical plate and subchondral cancellous bone beneath the calcified cartilage (Burr and Gallant, 2012). Under physiological conditions, the viscoelastic characteristic of articular cartilage allows the maximum contact area to avoid stress concentration when mechanical stress is loaded, and the subchondral plate and cancellous bone play a role in stress transmission (Burr and Gallant, 2012). In the early stage of OA, the thickness of the subchondral plate is reduced as bone remodeling is accelerated. In the late stage, subchondral bone sclerosis (hallmark of OA progression) occurs, and the increase of stiffness and density will lead to stress concentration and exacerbate overlying cartilage deterioration in return (Hu et al., 2021).

Sun X et al. (2020) discovered that miR-29b was downregulated in BMSCs extracted from condylar subchondral bone of the OA mouse model, and knockdown of this miRNA significantly rescued the subchondral bone loss and cartilage degradation, which means miR-29b functions as a vital mediator during articular cartilage and subchondral bone physiopathological change. As for members of the miR-17-92 cluster, it was reported that the expression level of miR-17-3p was low during BMP2-induced osteogenesis. SRY-box transcription factor 6 (SOX6), a transcription factor related to alteration of bone mineral density, was discovered to contain binding sites of miR-17-3p (Chen et al., 2020). The promotion effect of miR-17-3p inhibitor on osteoblast differentiation could be partially reversed by si-SOX6, which shows that miR-17-3p–SOX6 might be the candidate molecular mechanism for OA subchondral bone treatment. However, mechanisms that show how the miR-17-92 cluster is involved in the changes of OA subchondral bone turnover and its communication with overlying cartilage are still obscure.

## miR-17-92 in periarticular tissue destruction

### Synovitis

Synovitis predominant with T cells characterizes the autoimmune disease—rheumatoid arthritis (RA) (Giannini et al., 2020). As for OA, synovitis featured by macrophage infiltration emerges with disease progression and subsequently deteriorates the articular microenvironment to aggravate cartilage and subchondral bone lesions (Sellam and Berenbaum, 2010; Mathiessen and Conaghan, 2017).

Kuo et al. (2019) reported that treating OA synovial fibroblasts (OASFs) with TGF-β1 diminished the synthesis of TNF-α, IL-1β, vascular endothelial growth factor (VEGF), and C–C motif chemokine ligand 2 (CCL2) and stimulated forkhead box O3 (FOXO3) expression, which means FOXO3 mediates the TGF-β1-stimulated anti-inflammation effect. Bioinformatics prediction and functional experiments convinced that miR-92a was a molecule upstream of FOXO3, indicating that the inhibition of miR-92a was essential for FOXO3 release and thus alleviated OASF inflammation. It was reported that the secretion of chemokines (e.g., CCL2/MCP-1 and CCL3/MIP-1α) decreased when synoviocytes were co-cultured with micro-fragmented adipose tissue (MF) (Shi et al., 2022). Given that chemokines are crucial factors mediating monocytes/macrophage infiltration and subsequent inflammatory cytokine production in synovial tissues and fluids (Raghu et al., 2017), the aforementioned results indicate the potential treatment effects of MF on OA synovitis. Mechanistically, miR-92a-3p was detected to be downregulated in TNF-α-treated synoviocytes, and knockdown of miR-92a-3p overturned the protective effect of co-cultured MF upon synovitis (Shi et al., 2022), which contradicts the results from Kuo et al. (2019). Hence, more high-quality evidence is required to illustrate the roles of miR-92a in synovitis. In the rat OA model from another researcher (Feng et al., 2022), upon injecting adeno-associated virus (AAV)–miR-18a-3p, the proliferation of synovial membrane cells was inhibited, accompanied by the reduction of inflammatory cells.

With the OA progression, pro-inflammatory cytokines could affect periarticular ligaments, which contributes to symptoms like pain and articular immobility (Katz et al., 2021). Li et al. (2017) reported the miRNA microarray results of knee anterior cruciate ligaments (ACLs) with or without OA, showing that OA progression significantly downregulated the expression of miR-18a in ACL.

## miR-17-92 in OA treatment

### Stem cell therapy

Tissue engineering is a novel technology aimed at different kinds of tissue repair and regeneration with the compound of seed cells, biocompatible materials, and bioactive molecules (Yang et al., 2017; Armiento et al., 2018). During the development of these years, its great success and huge potential attract more and more researchers.

The global change of miRNA expression during chondrogenesis of human adipose-derived stem cells (hADSCs) was detected by microarray chips (Zhang et al., 2012). Hundreds of differentially expressed miRNAs were found after chondrogenic differentiation, among which miR-92a was upregulated. However, the results of aforementioned research studies were not extended to *in vivo* validation, which obscures the roles of miR-92a in stem cell therapy for the treatment of OA.

### Exogenous and endogenous miRNA promotion

Agomir/antagomir is usually used for promoting exogenous gene expression in experimental animal models. Agomir-17 injection reversed the high expression level of OA markers (i.e., MMP13, ADAMTS5, and nitric oxide synthase 2 (NOS2)) in mouse destabilization of the medial meniscus (DMM) model (Zhang B et al., 2022). Moreover, Lian et al. (2020) reported that in the combination of antagomir-18a and IL-1β inhibitor, better effects were observed on the suppression of chondrocyte hypertrophy and cartilage degradation than single anti-cytokine therapy, which means the injection of antagomir-18a sensitizes OA chondrocytes to anti-inflammatory cytokine and significantly promotes OA treatment.

From another research, AAV–miR-18a-3p was injected to promote exogenous miR-18a-3p expression in the rat knee OA model (Feng et al., 2022). Upon localized miR-18a-3p overexpression, the over-proliferation of synovial cells and the infiltration of inflammatory cells were significantly alleviated, and cytokines like IL-6 and PGE2, which are responsible for cartilage degradation and bone turnover deterioration, were in the low serum level in the miR-18a-3p group. To promote endogenous miR-17 expression, the intra-articular injection of GDF-5 was administered into the animal OA models (Zhang Y et al., 2022), which showed the significant effect of attenuating the knee OA phenotype.

### Extracellular vesicle

Extracellular vesicles (EVs) are nanoparticles carrying bioactive molecules (e.g., proteins, mRNA, and miRNA) with a lipid bilayer structure, and EVs can be categorized into three subtypes based on their biogenesis processes and surface markers (namely, microvesicles, exosome, and apoptotic bodies) (van Niel et al., 2018; Mathieu et al., 2019). Their release and transfer play a role in intercellular communication, extensively participating in pathophysiological situations, such as cancer, metabolic diseases, and inflammatory responses (Akbar et al., 2019; Harrell et al., 2019; Walker et al., 2019). Meanwhile, EV-based therapy is an ideal substitute for MSC-based tissue regeneration due to its advantage of avoiding tumorigenesis and immunological rejection (Nagelkerke et al., 2021). For instance, exosomal miR-320c from human bone marrow MSCs (hBMSCs) reversed SOX9 downregulation and MMP13 upregulation in OA chondrocytes, and such effects were enhanced by exosomes from miR-320c-overexpressed hBMSCs (Sun et al., 2019).

MSC exosomes (MSC-Exos) with or without chondrogenic differentiation were collected for miRNA microarray (Mao et al., 2018). It was detected that 141 miRNAs were differentially expressed, among which miR-92a-3p was nearly eight-fold higher in the chondrogenic group. miR-92a-3p-overexpressed MSC-Exos (MSC-miR-92a-3p-Exos) were found to promote chondrocyte proliferation and MSC chondrogenesis. With MSC-miR-92a-3p-Exo injection on 7, 14, and 21 days, OA-related cartilage matrix degradation was significantly alleviated, while MMP13 expression was reduced compared to the OA mouse model without treatment.

Zhang B et al. (2022) isolated EVs secreted by bone marrow stem cells (BMSCs); subsequent validation confirmed that they were approximately 150-nm oval particles with positive EV surface marker expression. After uptake of BMSC-EVs, the OA phenotype of chondrocytes was surprisingly relieved, and EVs collected from BMSCs incubated in a hypoxic environment showed even better therapeutic efficacy on inflammatory chondrocytes. In the rat OA model, injection of BMSC-EVs protected joint from cartilage degradation, and OARSI scores in the hypoxia-EV group was significantly lower than those in OA and OA + normal EV groups, which indicates the excellent OA-treating function of hypoxia-preconditioned EVs (Zhang Y et al., 2022). To clarify the bioactive contents in these nanoparticles, miRNA profiling was implemented and miR-18a-3p was found as one of the downregulated miRNAs in hypoxia-EVs (Zhang B et al., 2022) (Table 2).

**TABLE 2:** Treatment effect of the miR-17-92 cluster upon OA.

miRNA	Expression level	Function	Reference
miR-92a	Upregulated during hADSC chondrogenesis	-	Zhang et al. (2012)
miR-17	Downregulated in the mouse OA model	Its overexpression suppresses cartilage destruction *in vitro*, and local injection of agomir-17 inhibits OA progression *in vivo*	Zhang B et al. (2022)
miR-18a	Upregulated in the rat OA model	Antagomir-18a injection sensitizes chondrocytes to anti-inflammatory cytokine therapy	Lian et al. (2020)
miR-18a-3p	Downregulated in the IL-1β-induced OA model *in vitro*	AAV–miR-18a-3p injection inhibits synovial cell over-proliferation and inflammatory cell infiltration	Feng et al. (2022)
miR-92a-3p	Upregulated during MSC chondrogenic differentiation	miR-92a-3p-overexpressed MSC-exosomes promote chondrocyte proliferation *in vitro* and alleviate cartilage degradation *in vivo*	Mao et al. (2018)
miR-18a-3p	Downregulated in extracellular vesicles secreted by BMSCs	BMSC-exosome featured with low level miR-18a-3p alleviates OA inflammation *in vitro* and *in vivo*	Zhang Y et al. (2022)

hADSCs, human adipose-derived stem cells; OA, osteoarthritis; BMSCs, bone marrow mesenchymal stem cells.

## Conclusion

The onset and progression of OA are complex processes related to multiple tissues—cartilage, subchondral bone, synovium, and periarticular ligament. The pathological transformation of such tissues will affect each other, thus deteriorating the microenvironment in a vicious circle. miRNAs are one of the best understood mediators that epigenetically regulate physiological and pathological activities across species. As one miRNA could target a great number of downstream genes and a single mRNA could be complementarily blocked by various miRNAs, the multifaceted function and regulatory network would be complex even if functional redundancy exists. Although the participation of the miR-17-92 cluster in OA was extensively explored, the regulatory network construction of the six constituents remains a lot of work to perform.

As for the whole picture of the miR-17-92 cluster, several problems are still required for clarification. First, researchers up to date mostly focus on the roles of miR-17-92 in a single tissue of OA joint, and little is known about communication between different articular tissues (e.g., overlying cartilage and subchondral bone; synovium and cartilage), which is vital for joint homeostasis and equally important in joint diseases. Thus, more investigations are required to explore the function of the miR-17-92 cluster during such cross-talk in the future. Then, a well-designed *in vivo* experiment and a medication delivery system are still rare in miR-17-92-mediated treatment. In current *in vivo* research, the miR-17-92 cluster is usually delivered through viral vectors like AAVs; viral vectors have the advantage of excellent delivery efficiency, but they might cause tumorigenesis and immune rejection. To make it safe enough to deliver miR-17-92 into OA joint in human, developing ideal non-viral delivery systems could be a good choice. Moreover, there still exists contradiction concerning the roles of miR-17-92 in OA. One single miRNA could show reverse effect on the OA progression in different literature. For example, Kuo et al. (2019) reported that the inhibition of miR-92a could alleviate the inflammation of OA synovial fibroblasts, but another research discovered that miR-92a was downregulated in TNF-α-treated synoviocytes (Shi et al., 2022). The reason of such phenomena might be the differences in cell lineage and experimental conditions. To better illustrate the underlying mechanisms and current contradictions, more high-quality research studies about this topic are required in the future.
